# Conicity index as an indicator of abdominal obesity in individuals with chronic kidney disease on hemodialysis

**DOI:** 10.1371/journal.pone.0284059

**Published:** 2023-04-19

**Authors:** Cleodice Alves Martins, Camila Bruneli do Prado, Júlia Rabelo Santos Ferreira, Monica Cattafesta, Edson Theodoro dos Santos Neto, Fabiano Kenji Haraguchi, José Luiz Marques-Rocha, Luciane Bresciani Salaroli

**Affiliations:** 1 Graduate Program in Nutrition and Health, Health Sciences Center, Federal University of Espírito Santo, Vitória, Espírito Santo, Brazil; 2 Graduate Program in Collective Health, Health Sciences Center, Federal University of Espírito Santo, Vitória, Espírito Santo, Brazil; Health Sciences University, Faculty of Medicine, Bursa City Health Practice and Research Center, TURKEY

## Abstract

**Background:**

The conicity index is indicated as a tool for assessing the nutritional status of renal individuals undergoing hemodialysis. Thus, this study aimed to estimate the prevalence of abdominal obesity using the conicity index in individuals with chronic kidney disease undergoing hemodialysis to verify its association with sociodemographic, clinical, and lifestyle factors.

**Materials and methods:**

This is a cross-sectional study with 941 individuals undergoing hemodialysis in a metropolitan area in southeastern Brazil. The conicity index was estimated and cutoffs of 1.275 and 1.285 for men and women, respectively, were used. For the analysis of the results, binary logistic regression was performed and the odds ratio (OR) was estimated with their respective confidence intervals (95% CI).

**Results:**

The conicity index was high in 56.54% of men (95% CI: 34.34–70.16) and 43.46% of women (95% CI: 38.45–55.20). We found that both adult men (OR = 3.71; 95% CI: 2.27–6.07) and adult women (OR = 4.06; 95% CI: 2.41–6.84) were more likely to have abdominal obesity, as well as self-declared mixed-raced (OR: 1.74; 95% CI: 1.01–3.00) and single men (OR: 1.64; 95% CI: 1.00–2.68).

**Conclusions:**

The conicity index is an important anthropometric indicator to estimate abdominal obesity in individuals with chronic kidney disease on hemodialysis.

## Introduction

The prevalence of end-stage renal disease, which is increasingly growing worldwide, reflects the environmental, lifestyle, and sociodemographic particularities of each country [1, 2]. Dialysis is the predominant renal replacement therapy in most countries [1], with hemodialysis being the most common modality, covering 90.5% of individuals with chronic kidney disease (CKD) in Brazil [3].

In parallel, the increasing incidence of obesity in individuals on hemodialysis is a challenge to public health [4–6], as it is associated with several complications including cardiometabolic disorders [7, 8], contraindication to kidney transplantation [9], insulin resistance, and increased mortality [10–12]. Regarding body fat distribution, studies show that abdominal obesity results in worse clinical outcomes in this population such as reduced lean mass, high levels of inflammation, lower physical capacity [6], and higher mortality [13]. It is also a significant predictor of cardiovascular events [14], regardless of body mass index (BMI).

In this context, the use of anthropometric indicators proves to be a viable method due to its simplicity, low operational cost, speed, and effectiveness in assisting in the monitoring and early diagnosis of abdominal obesity, especially in individuals with chronic kidney disease (CKD) on hemodialysis [15, 16]. Among them, the application of the conicity index is recommended by the guideline of clinical practice for nutrition in individuals with CKD on hemodialysis as tracking of nutritional status and predictor of mortality [17].

The conicity index was proposed by Valdez [18] aiming to identify abdominal obesity and is determined using measurements of body mass, height, and waist circumference. It is based on the distribution of body fat, which by accumulating fat in the abdominal region, changes the body from a cylindrical shape (without accumulation of abdominal fat) to a double-cone shape with a common base (accumulation of abdominal fat). Thus, individuals with a high conicity index have abnormal fat deposition in the abdominal region in relation to their height and weight. The indicator is recognized as an efficient tool [19] for identifying visceral fat [20–22], cardiovascular risk [23], lipid alterations [24], and coronary risk [25].

However, studies that diagnose the presence of abdominal obesity by the conicity index do not use a specific cutoff point for the index for individuals with CKD on hemodialysis. In previous studies, a specific cutoff point was developed for the conicity index for renal individuals [26], which was applied in the present study considering the importance of investigating the distribution of body fat in this population. This article aimed to estimate the prevalence of abdominal obesity using the conicity index in individuals with chronic kidney disease undergoing hemodialysis to verify its association with sociodemographic, clinical, and life habits factors.

## Materials and methods

### Study population

This is a cross-sectional, analytical, and epidemiological study conducted in 11 hemodialysis centers in a metropolitan area in southeastern Brazil.

Data collection for the original study took place from February to September 2019. The population of this study consisted of 941 individuals. Participants were eligible for inclusion when diagnosed with CKD, of both genders, over 18 years, and on hemodialysis for at least six months. Individuals with contact precautions, language barriers, edema, ascites, and with missing data on the outcome were excluded from the study.

### Sociodemographic and clinical characteristics

The variables skin color, income, marital status, work activity, and schooling level were self-declared. The time of the diagnosis of chronic kidney disease and hemodialysis treatment were collected from medical records. Income was defined as the minimum wage, which is the lowest salary a company must pay to an employee according to the number of hours worked and is reassessed annually based on the cost of living in Brazil. In the year that the study was conducted, the minimum wage was R$998.00, or US$235,37.

The categorization of physical activity is described in greater detail in the previous study [26]. Regarding smoking, those who reported being smokers were categorized as “yes” and those who reported never having smoked or who had smoked in the past were categorized as “no.” Regarding alcohol intake, those who reported consuming alcohol, regardless of time or quantity, were classified as “yes,” and those who reported not consuming alcoholic beverages were classified as “no.”

### Anthropometry

The anthropometric evaluation was performed according to the recommended protocols [27] after the hemodialysis session by trained professionals and with the use of standardized and calibrated equipment. The individuals were weighed using a portable digital electronic scale (Tanita^®^, São Paulo, Brazil) and their height was measured using a portable stadiometer (Sanny^®^, São Paulo, Brazil). Participants were instructed to remain in an upright position, barefoot, wearing as little clothing as possible, and with arms extended along the body. Waist circumference (WC) was assessed using an inelastic measuring tape (Sanny®, São Paulo, Brazil) positioned at the midpoint between the lower edge of the costal arch and the iliac crest. All anthropometric measurements were performed three times to obtain the arithmetic mean.

From the anthropometric measurements, the BMI and the conicity index were estimated. BMI was obtained by dividing weight (kg) by height squared (m^2^), and we adopted the classification for adults and older adults according to the WHO [28]. The conicity index was estimated from weight, height, and WC measurements using the following equation:

$$\frac{\mathrm{Waist}\;\mathrm{circumference}\;(m)}{0.109\sqrt{\frac{\mathrm{Weight}\;(\mathrm{kg})}{\mathrm{Height}\;(m)}}}$$

In this study, the cutoff points adopted for the conicity index for individuals with CKD on hemodialysis were ≥ 1.275 for men and ≥ 1.285 for women [26]. To make the results consistent, the cut-off point applied in the study was performed with individuals on hemodialysis, as this population has specific body differences generated by chronic kidney disease [6, 7, 10, 12, 13], which makes it important to use a specific cut-off point for the index of conicity [26].

### Statistical analysis

The descriptive analysis was stratified by gender by evaluating the presence or absence of abdominal obesity using the conicity index, with categorical variables presented through relative and absolute frequencies and numerical variables presented through the median and interquartile ranges. The normality of the variables was evaluated using the Kolmogorov-Smirnov test. The Mann-Whitney test was used to compare the medians. Pearson’s chi-square test (x^2^) was used for qualitative variables in the tests of association between the independent variables and the outcome.

The binary logistic regression model was used to evaluate associations between the independent variables and the conicity index, including in the model the variables that presented a p-value ≤ 0.10 in the bivariate analysis. For all of them, the assumptions of an absence of multicollinearity were evaluated (tolerance > 0.1 and variance inflation factor < 10), the minimum sample size for the number of variables in the model (> 20 individuals per variable in the model and > 5 cases in each category of variables), and the absence of outliers. For the binary logistic regression analysis, the enter method was used, adopting the model with the highest adjustment according to the Nagelkerke test (p>0.05, closer to 1.0). All analyses were conducted in the R program (4.0.3) for Windows. The significance level adopted was 5%.

### Ethical aspects

This study was approved by the Research Ethics Committee no. 4.023.221 (CAAE 68528817.4.0000.5060) and the Informed Consent Form was obtained, in which all signed and gave their consent for the research to be carried out.

## Results

Of the 1,351 hemodialysis users present at the time of data collection, 304 were excluded for not meeting the inclusion criteria, 23 for refusing to participate in the study, and 83 for lacking outcome data. Of these, 532 men (56.54%; 95% CI: 34.34–70.16) and 409 women (43.46%; 95% CI: 38.45–55.20) have abdominal obesity, totaling 615 individuals (65.35%; 95% CI: 50.75–77.10) according to the conicity index. The median conicity index of the 941 subjects included in this study showed a significant difference between genders (p<0.016), with men having the highest median (interquartile range [IQR]) of 1.32 (IQR: 1.25–1.40) compared to women (IQR of 1.31) (IQR: 1.22–1.38).

Table 1 presents the bivariate analysis of the conicity index stratified by gender considering sociodemographic, clinical, and lifestyle variables. We found proportional differences between age (p<0.001), ethnicity/skin color (p<0.001), income (p = 0.003), marital status (p<0.001), work activity (p = 0.010), and BMI (p<0.001) in men. In women, the variables that showed statistical difference were age (p<0.001), marital status (p = 0.003), work activity (p<0.001), and BMI (p<0.001).

**Table 1:** Comparison of sociodemographic and clinical indicators and lifestyle habits according to the conicity index stratified by sex of individuals on hemodialysis.

Variables	Men			Women		
	CI < 1.275	CI ≥ 1.275	p-value*	CI < 1.285	CI ≥ 1.285	p-value*
	163 (17.32%)	369 (39.21%)		163 (17.32%)	246 (26.14%)	
**Age (years)**			**<0.001**			**< 0.001**
20 to 59	133 (25.00%)	171 (32.14%)		129 (31.54%)	120 (29.34%)	
60 or more	30 (5.64%)	198 (37.22%)		34 (8.31%)	126 (30.80%)	
**Race/Color**			**<0.001**			0.725
White	26 (4.88%)	126 (23.68%)		42 (10.27%)	57 (13.93%)	
Brown	92 (17.30%)	154 (28.95%)		83 (20.30%)	135 (33.00%)	
Black	45 (8.46%)	89 (16.73%)		38 (9.29%)	54 (13.20%)	
**Income (MW)**			**0.003**			0.202
≤ 1	27 (5.20%)	33 (6.36%)		23 (5.90%)	21 (5.38%)	
> 1 a 2	71 (13.68%)	132 (25.43%)		72 (18.46%)	127 (32.56%)	
> 2 a 5	46 (8.86%)	121 (23.31%)		50 (12.82%)	65 (16.66%)	
> 5	17 (3.28%)	72 (13.88%)		12 (3.07%)	20 (5.13%)	
**Marital Status**			**<0.001**			**0.003**
Married/Lives with partner	89 (16.73%)	254 (47.75%)		68 (16.62%)	116 (28.36%)	
Divorced/Widowed	17 (3.19%)	55 (10.34%)		40 (9.77%)	82 (20.04%)	
Unmarried	57 (10.71%)	60 (11.28%)		55 (13.44%)	48 (11.73%)	
**Work Activity**			**0.010**			**0.001**
No paid work activity	72 (1.13%)	215 (40.80%)		42 (10.47%)	52 (12.96%)	
With paid work	83 (15.75%)	143 (27.13%)		52 (12.96%)	51 (12.71%)	
Retired or on sick leave	6 (13.66%)	8 (1.53%)		63 (15.71%)	141(35.16%)	
**Schooling (years)**			0.146			0.086
< 8	55 (10.40%)	147 (27.79%)		59 (14.64%)	113 (28.04%)	
≥ 8 to < 11	92 (17.40%)	173 (32.70%)		83 (20.60%)	98 (24.31%)	
≥ 11	16 (3.02%)	46 (8.70%)		20 (4.96%)	30 (7.44%)	
**CKD diagnosis (years)**			0.546			0.453
≤ 1	30 (5.67%)	75 (14.18%)		25 (6.14%)	47 (11.55%)	
> 1 a 5	65 (12.29%)	162 (30.62%)		60 (14.74%)	97 (23.83%)	
> 5 a 10	39 (7.37%)	77 (14.55%)		44 (10.81%)	51 (11.56%)	
> 10	29 (5.48%)	52 (9.83%)		33 (8.11%)	50 (12.28%)	
**Hemodialysis time (years)**			0.188			0.232
≤ 1	31 (6.19%)	76 (15.17%)		27 (6.94%)	59 (15.16%)	
> 1 a 5	61 (12.17%)	158 (31.53%)		63 (16.19%)	96 (24.68%)	
> 5 a 10	32 (6.39%)	64 (12.77%)		38 (9.77%)	45 (11.56%)	
> 10	32 (6.39%)	47 (9.38%)		27 (6.94%)	34 (8.74%)	
**Smoking**			0.529			0.167
No	155 (29.13%)	344 (64.67%)		153 (37.41%)	239 (58.43%)	
Yes	8 (1.50%)	25 (4.70%)		10 (2.44%)	7 (1.71%)	
**Alcohol Intake**			0.212			0.656
No	137 (25.81%)	328 (61.70%)		154 (37.65%)	236 (57.70%)	
Yes	25 (4.71%)	41 (7.78%)		9 (2.20%)	10 (2.44%)	
**Physical activity**			0.176			0.140
Dont’t practice	107 (20.15%)	269 (50.66%)		132 (32.28%)	213 (52.07%)	
Below recommended	29 (5.46%)	45 (8.47%)		14 (3.42%)	20 (4.89%)	
Withing the recommended	26 (4.90%)	55 (10.36%)		17 (4.15%)	13 (3.19%)	
**BMI (kg/m^2^)**			**<0.001**			**<0.001**
< 18.5	15 (2.85%)	4 (0.76%)		20 (4.89%)	1 (0.24%)	
18.5 to 24.9	122 (23.10%)	140 (26.51%)		100 (24.44%)	77 (18.82%)	
25 to 29.9	21 (3.98%)	152 (28.78%)		13 (7.33%)	89 (19.31%)	
> 30	4 (0.76%)	70 (13.26%)		30 (3.18%)	79 (21.76%)	

CI, Conicity Index; CKD, Chronic Kidney Disease; BMI, Body Mass Index; MW, minimum wage.

*Chi square test

Eutrophic individuals diagnosed by BMI already had abdominal obesity indicated by the conicity index, which corresponded to 144 (27.27%) in men and 78 (19.06%) in women, totaling almost half of the study population (46.33%).

Table 2 shows binary logistic regression between the independent variables and abdominal obesity stratified by the male gender. The variables age–adult (OR: 3.71; p<0.001; 95% CI: 2.27–6.07). ethnicity/ skin color–Brown (OR: 1.74; p = 0.043; 95% CI: 1.01–3.00). and marital status–single (OR: 1.64; p = 0.047; CI 95%: 1.00–2.68) showed an increased chance of abdominal obesity.

**Table 2:** Binary logistic regression between associated variables in the bivariate analysis and the outcome.

Variables	Crude		Adjusted	
	p-value	OR (95% CI)	p-value*	OR (95% CI)
**Age (years)**				
20 to 59	<0.001	3.82 (2.35–6.20)	**<0.001**	**3.71 (2.27–6.07)**
60 or more		1		**1**
**Race/Color**				
White		1		**1**
Brown	0.012	1.97 (1.15–3.35)	**0.043**	**1.74 (1.01–3.00)**
Black	0.068	1.73 (0.95–3.12)	0.128	1.59 (0.87–2.92)
**Marital Status**				
Married/Lives with partner		1		1
Divorced/Widowed	0.770	0.91 (0.48–1.71)	0.670	0.86 (0.45–1.65)
Unmarried	0.018	1.78 (1.10–2.90)	**0.047**	**1.64 (1.00–2.68)**
**Work Activity**				
No paid work activity		1		1
With paid work	0.573	1.39 (0.44–4.40)	0.408	1.66 (0.49–5.58)
Retired or on sick leave	0.751	1.20 (0.37–3.89)	0.588	1.40 (0.41–4.79)

OR, odds ratio; 95% CI, 95% Confidence Interval.

* Nagelkerke fit quality: 0.593

Regarding women. being an adult (OR: 4.06; p<0.001; 95% CI: 2.41–6.84) increased by approximately four times the chance of presenting a high conicity index (Table 3).

**Table 3:** Binary logistic regression between associated variables in the bivariate analysis and the outcome.

Variables	Crude		Adjusted	
	p-value	OR (95% CI)	p-value*	OR (95% CI)
**Age (years)**				
20 to 59	<0.001	3.48 (2.14–5.67)	**<0.001**	**4.06 (2.41–6.84)**
60 or more		1		1
**Marital Status**				
Married/Lives with partner		1		1
Divorced/Widowed	0.349	1.28 (0.75–2.18)	0.211	1.43 (0.81–2.52)
Unmarried	0.169	1.44 (0.85–2.45)	0.197	1.44 (0.85–2.45)
**Work Activity**				
No paid work activity		1		1
With paid work	0.683	1.13 (0.62–2.03)	0.917	1.03 (0.55–1.93)
Retired or on sick leave	0.108	0.64 (0.37–1.10)	0.104	0.61 (0.34–1.10)
**Schooling (years)**				
< 8		1		1
8 to 11	0.398	1.22 (0.76–1.96)	0.836	1.05 (0.63–1.74)
> 11	0.985	1.00 (0.48–2.03)	0.523	0.76 (0.33–1.74)

OR, odds ratio; 95% CI, 95% Confidence Interval.

* Nagelkerke fit quality: 0.517

## Discussion

This is the first study to evaluate abdominal obesity according to a cutoff point of the specific conicity index for individuals with CKD on hemodialysis. The conicity index is a good indicator of fat distribution capable of capturing variations in body composition and allowing comparisons between subjects who have different measurements of body weight and height [29].

Studies demonstrate the strong correlation of the conicity index with the detection of visceral fat using computed tomography [12, 20–22]. However imaging tests such as computed tomography and magnetic resonance imaging are expensive and require a specialized team. Thus the conicity index is an accurate, simple and low-cost alternative that can be used as a substitute for more sophisticated tests to detect abdominal obesity [12, 20, 21].

Regarding age group being an adult increases the chances of having abdominal obesity in both men and women which represents a higher risk of all-cause mortality for this population [13]. Although the cutoff point for the indicator is higher for women the prevalence of abdominal obesity in this study was higher among men.

Women with CKD on hemodialysis are more likely to have a higher conicity index than men with a median above 1.5 however the highest percentage of abdominal obesity was observed in men [30]. Previous studies clarify that men have greater increases in central adiposity than women conferring the android fat distribution format [31, 32].

The distribution of abdominal fat differs between genders. Although women tend to have lower body mass fat mass tends to be greater than in men [33]. Women have an accumulation of adipose tissue in the hips and thighs called gynoid fat [34]. Excess general adiposity or increased central obesity are predictors of an increased risk of cardiovascular and cerebrovascular complications [35] and anthropometric measurements are better predictors of increased cardiovascular risk compared to measures of general obesity such as BMI [36].

An interesting result in our study was the diagnosis of eutrophy or low weight by using BMI in parallel with the presence of abdominal obesity in some individuals. In general BMI is a standardized anthropometric measure well accepted nationally and internationally for defining obesity in clinical practice and research [37–39]. However studies show the fragility of the evaluation of nutritional status for individuals on hemodialysis since it ignores the distribution of body fat or the distinction between fat mass and lean mass [40]. Therefore the literature suggests that the analysis of body fat distribution can be more meaningful than overall adiposity [12, 13, 41–43].

Low BMI concomitant with increased central adiposity results in an increased risk of all-cause mortality in this population [13] as well as mortality from cardiovascular disease [14, 44]. Yeganehjoo et al. [45] demonstrated that underweight individuals with CKD on hemodialysis had the highest conicity index compared to eutrophic and overweight groups suggesting an increase in central adiposity. These results corroborate our findings.

Furthermore another study showed [6] that the presence of abdominal obesity despite adequate BMI results in worse clinical outcomes including reduced lean body mass, high levels of inflammation and lower physical capacity compared to individuals with CKD who have a BMI with excess weight and the presence of abdominal obesity. Therefore using anthropometric methods other than BMI to assess central adiposity is beneficial.

Regarding ethnicity/skin color we demonstrated that among men mixed-raced people are more likely to have abdominal obesity. Some studies have found similar results regarding non-white groups [46, 47] showing that Black/Mixed-raced men and women have a greater amount of abdominal fat than Whites [48]. But we should emphasize the prevalence of the Black and mixed-raced population in this study which may justify the results found.

We found that the marital status of individuals may reflect on the distribution of body fat since having a partner tends to awaken favorable life habits including understanding their clinical situation and following dietary guidelines [49, 50]. Married individuals or those with a partner are more physically active [50] and those with CKD on hemodialysis may have better support and understanding of their health and application in practice. Those without a partner can benefit from the support of their social circle, multidisciplinary education and care plans adjusted to the situation of their patients [50].

Our study is limited by its cross-sectional nature without any manipulation of exposure factors consequently not assessing causal effects related to the analyzed outcome. In addition there may be difficulties in calculating the denominator of the proposed equation for determining the conicity index in population studies. For this reason a study carried out in a Brazilian population produced a table [25] which by crossing information on weight and height it is possible to verify the denominator value of the Conicity Index being only necessary to divide the waist circumference value (in meters) with the denominator value.

However to the best our knowledge. this study was the first to conduct the association with sociodemographic, clinical and lifestyle factors using a cutoff point of the specific conicity index for individuals with CKD undergoing hemodialysis. Moreover data on medical history were confirmed by medical records filled out longitudinally in hemodialysis services and compared to information provided to researchers by self-report.

## Conclusions

Our results confirm the presence of abdominal obesity indicated by the conicity index regardless of the eutrophic state diagnosed by BMI in addition to the association of the indicator with adult age group, ethnicity/ skin color and marital status. Therefore we suggest using the conicity index as a simple and efficient tool for tracking abdominal obesity to provide significant information about the nutritional status of individuals with CKD on hemodialysis.
